# Controlling Extra- and Intramacrophagic *Mycobacterium abscessus* by Targeting Mycolic Acid Transport

**DOI:** 10.3389/fcimb.2017.00388

**Published:** 2017-09-01

**Authors:** Albertus Viljoen, Jean-Louis Herrmann, Oluseye K. Onajole, Jozef Stec, Alan P. Kozikowski, Laurent Kremer

**Affiliations:** ^1^Centre National de la Recherche Scientifique UMR9004, Institut de Recherche en Infectiologie de Montpellier, Université de Montpellier Montpellier, France; ^2^UMR1173, INSERM and UFR Des Sciences de la Santé Simone Veil, Université de Versailles Saint Quentin Montigny-le-Bretonneux, France; ^3^Department of Biological, Chemical and Physical Sciences, Roosevelt University Chicago, IL, United States; ^4^Department of Pharmaceutical Sciences, College of Pharmacy, Marshall B. Ketchum University Fullerton, CA, United States; ^5^StarWise Therapeutics LLC, University Research Park Madison, WI, United States; ^6^INSERM, IRIM, 34293 Montpellier, France

**Keywords:** *Mycobacterium abscessus*, macrophage, glycopeptidolipid, mycolic acid, MmpL3, chemotherapy

## Abstract

*Mycobacterium abscessus* is a rapidly growing mycobacterium (RGM) causing serious infections especially among cystic fibrosis patients. Extremely limited therapeutic options against *M. abscessus* and a rise in infections with this mycobacterium require novel chemotherapies and a better understanding of how the bacterium causes infection. Different from most RGM, *M. abscessus* can survive inside macrophages and persist for long durations in infected tissues. We recently delineated differences in the infective programs followed by smooth (S) and rough (R) variants of *M. abscessus*. Unexpectedly, we found that the S variant behaves like pathogenic slow growing mycobacteria, through maintaining a block on the phagosome maturation process and by inducing phagosome-cytosol communications. On the other hand, R variant infection triggers autophagy and apoptosis, reminiscent of the way that macrophages control RGM. However, the R variant has an exquisite capacity to form extracellular cords, allowing these bacteria to rapidly divide and evade phagocytosis. Therefore, new chemotherapeutic interventions against *M. abscessus* need to efficiently deal with both the reservoir of intracellular bacilli and the extracellular cords. In this context, we recently identified two chemical entities that were very effective against both *M. abscessus* populations. Although being structurally unrelated these two chemotypes inhibit the activity of the essential mycolic acid transporter, MmpL3. In this Perspective, we aimed to highlight recent insights into how *M. abscessus* interacts with phagocytic cells and how the inhibition of mycolic acid transport in this pathogenic RGM could be an efficient means to control both intracellular and extracellular populations of the bacterium.

## Introduction

*Mycobacterium abscessus* is a rapidly growing mycobacterium (RGM) increasingly acknowledged as a serious non-tuberculous mycobacterial (NTM) pathogen (Mougari et al., 2016; Diel et al., 2017). Although it can cause extrapulmonary infections (Jeong et al., 2017) as well as disseminated pulmonary disease among otherwise healthy individuals (Varghese et al., 2012), it has become notorious for the serious threat it poses to cystic fibrosis (CF) patients. For these patients, *M. abscessus* infection is correlated with a decline in pulmonary function as well as challenges during last-resort lung transplantation (Esther et al., 2010; Smibert et al., 2016). *M. abscessus* exhibits high intrinsic resistance to many antibiotics making infections with this mycobacterium hard to treat (van Dorn, 2017). The macrolide drug clarithromycin has proven a relatively efficient treatment for *M. abscessus* infections, but high resistance implicating mutations in the *23S rRNA* gene and inducible resistance through the *erm(41)* gene, often result in clinical failures (Bastian et al., 2011). The few other antibiotics available include amikacin, linezolid and the β-lactams cefoxitin and imipenem, although the presence of a broad spectrum β-lactamase in the *M. abscessus* genome poses an obstacle to the use of these antibiotics, leading to the recommendation that these be co-administred with a β-lactamase inhibitor (Dubée et al., 2015).

*M. abscessus* presents distinct smooth (S) and rough (R) colony morphotypes, which is determined by the presence (S) or absence (R) of cell wall surface associated glycopeptidolipids (GPL) (Medjahed et al., 2010). The S variant is thought to be the colonizing form and is capable of producing mature biofilms and also has the ability to slide on soft agar (Howard et al., 2006). The R variant on the other hand is impaired in these abilities, but is capable of forming exquisite serpentine cords, a feature which is associated with its hypervirulence compared to the S form (Howard et al., 2006; Bernut et al., 2014). Phylogenetically, the *M. abscessus* complex consists of three sub-species, *M. abscessus* subsp. *abscessus, M. abscessus* subsp. *massiliense*, and *M. abscessus* subsp. *bolletii*, presenting different susceptibility profiles to clarithromycin and hence leading to different clinical outcomes (Jeong et al., 2017; Park et al., 2017). Being the most pathogenic RGM, it is not surprising that *M. abscessus* resists killing by phagocytic cells such as macrophages, a trait shared with its more generally pathogenic slow growing mycobacterium (SGM) relatives, such as *M. tuberculosis, M. bovis*, and *M. leprae* (Byrd and Lyons, 1999; Oberley-Deegan et al., 2009; Nessar et al., 2011).

Herein, we will first detail our recent findings highlighting the distinct intracellular fates of S and R *M. abscessus* and how these observations bring new insights into the lifestyle of the bacterium during acute and chronic phases of infection. In the second part, we discuss the recent discovery of compounds targeting mycolic acid transport in the bacterium, which are equally efficient on extracellular and intracellular bacteria, on S and R forms.

### The intracellular lifestyle of *M. abscessus*

**Figure 1:**
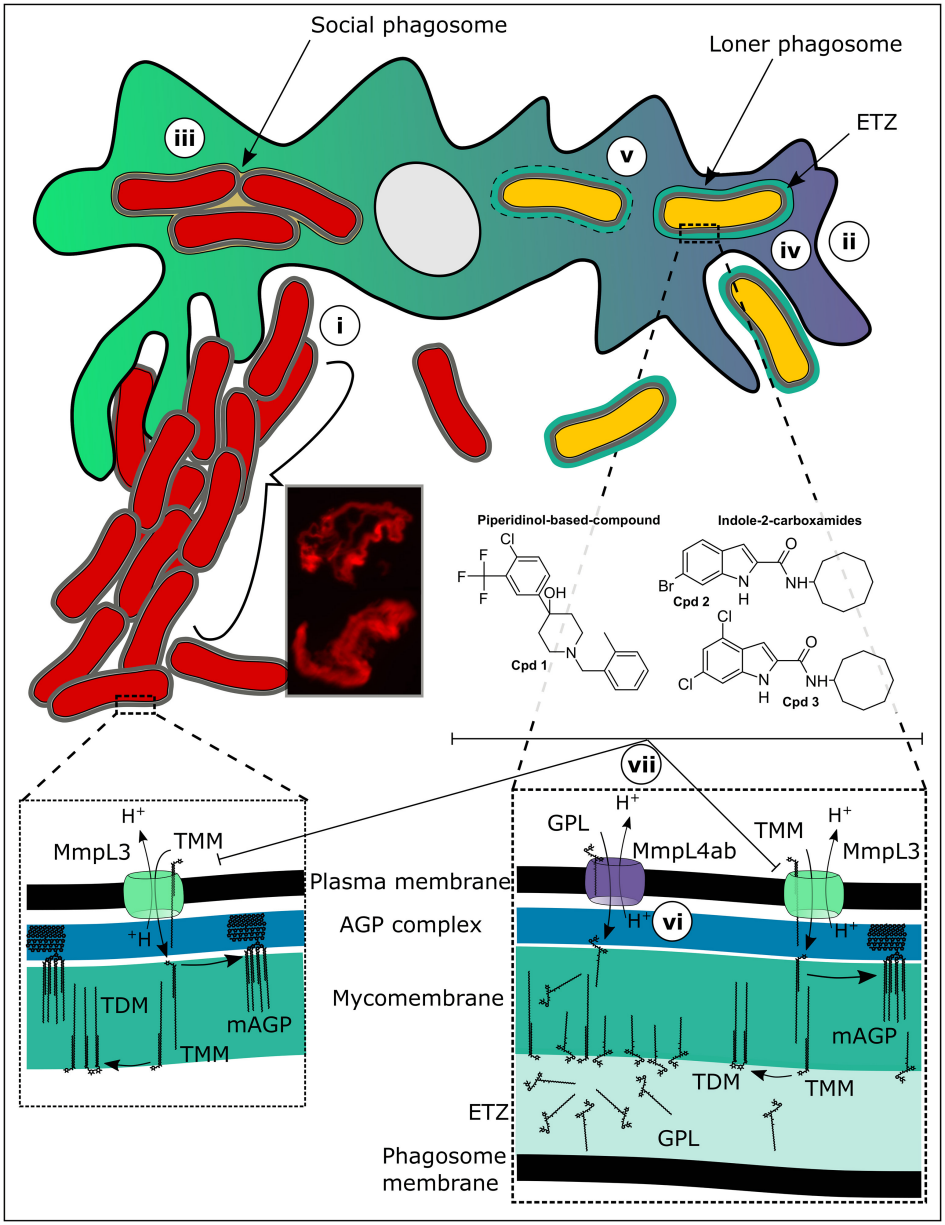
Targeting mycolic acid transport in extracellular and intracellular *M. abscessus*. **(i)** While rough (R) *M. abscessus* aggressively clumps and grows as serpentine cords in the extracellular milieu, evading phagocytosis by macrophages, **(ii)** smooth (S) *M. abscessus*, which is present as mostly singular organisms in the extracellular milieu, is easily phagocytosed. Once inside macrophages **(iii)** the R form is present in large social phagosomes containing numerous bacteria. These phagosomes mature rapidly and fuse with lysosomes. However, despite the acidic and radical environment present within these phagolysosomes, the R variant continues to divide rapidly, overpowering the macrophage defenses and resulting in autophagy and apoptosis. The S variants on the other hand **(iv)** remain in immature phagosomes because a tight apposition of their cell walls is maintained all around with the phagosomal membrane. These bacteria are not toxic to the cells and do not impact greatly upon the survival of the infected macrophages. The S form then **(v)** induces phagosome-cytosol communications through disruptions in the phagosome membrane providing access to cytosolic nutrients potentially, chronically sustaining a small population of persistent bacteria. Another feature of the S form-containing phagosomes is the large electron translucent zone (ETZ) which, is observable by electron microscopy and which is almost completely absent in R form-containing phagosomes. The ETZ which is a large outermost part of the mycobacterial cell wall is dependent upon the presence of large quantities of glycopeptidolipids (GPL). Mutations, for example resulting in amino acid substitutions in critical residues of the protein MmpL4a **(vi)**, which transports GPL from the cytosolic face of the bacterial plasma membrane where they are made to the outer membrane, result in the disappearance of a prominent ETZ. Inhibition of the mycolic acid transporter, MmpL3 **(vii)**, by a piperidinol-based derivative (compound 1) or indole-2-carboxamides (compounds 2 and 3), leads to abrogation of arabinogalactan mycolylation and of the production of trehalose dimycolate (TDM). This efficiently stops the growth of both intracellular and extracellular *M. abscessus*.

That the S and R forms of *M. abscessus* have different survival profiles in human monocytes was first reported by Byrd and Lyons (1999). While the S form survived poorly in human monocyte monolayers, the R form persisted. This result was later reproduced by independent studies, including our own (Howard et al., 2006; Nessar et al., 2011; Roux et al., 2016). Our observations of the sub-cellular events that exemplify infection with *M. abscessus* S or R forms (Figure 1) suggested that infection with the R form was reminiscent of an RGM infection, while that with the S form was more similar to an SGM infection (Roux et al., 2016).

To explain this dichotomy, we will first discuss what happens when the R variant encounters phagocytes. The R variant, which is highly aggregative in nature forms clumps in culture that are very hard to break using gentle techniques such as short bursts of sonication or passing the bacteria through a syringe needle (Bernut et al., 2015). It is thus very hard obtaining homogenous suspensions of the R variant and small clumps are practically impossible to avoid when infecting macrophage monolayers. As a consequence, macrophage cells engulf clumps of R bacteria resulting in large phagosomes containing numerous bacilli, quite different from the case for the S variant for which it is easier to prepare single cell suspensions (Roux et al., 2016). This effect of clumping of the *M. abscessus* R variant on infection of macrophages *in vitro* was the subject of a recent study (Brambilla et al., 2016) where phagocytosis of clumps of R bacteria resulted in rapid death of the infected J774 macrophages, while macrophages infected with the S variant stayed viable throughout the course of the infection. In our study, we observed similar events for infection of murine bone marrow-derived macrophages (BMDM). Under the transmission electron microscope, we observed large clumps of R variant bacteria lodged in phagocytic cups still on the exterior of the macrophage cells, either just prior to being phagocytosed or prohibiting their phagocytosis because of their sheer size (Roux et al., 2016). Once inside phagosomes, the R forms were rarely present as singular bacteria (loner phagosomes), but were found in groups of two or more bacilli per phagosome (social phagosomes) (Figure 1). These phagosomes contained lysosomal material probably because the close apposition between the mycobacterial cell walls and the phagosome membrane is interrupted in the social phagosome, which results in repression of the phagosome maturation block (de Chastellier et al., 2009). However, despite the presence of lysosomal material in these social phagosomes, the bacteria did not appear damaged, suggesting that they rescued themselves from the phagolysosomes (de Chastellier et al., 2009). In line with these results, R variant-containing THP1 macrophages were more acidified than those infected with the S variant and were autophagic and apoptotic, traits resembling infection with an RGM (Bohsali et al., 2010).

In contrast, the S variant behaved quite differently, where S forms were generally phagocytosed individually and also occurred most of the time in loner phagosomes (Roux et al., 2016). In these phagosomes, a tight apposition was maintained between the phagosome membrane and the bacterial cell wall all around. As a result, the phagosome maturation block was maintained and no lysosomal material was observed within these phagosomes. This result was supported by an absence of acidification observed in THP1 cells infected with the S form. Another striking feature of the S variant containing phagosomes was the presence of a large electron translucent zone (ETZ) surrounding the bacteria. The ETZ is a major part of the outer layer of the mycobacterial cell wall (Draper, 1974). On the contrary, the ETZ was barely visible in R variant containing phagosomes, as well as in phagosomes containing *M. abscessus* in which the gene encoding MmpL4b, a component of GPL synthesis and transport machinery, was deleted (Figure 1). In an independent study, we obtained similar results for *M. bolletii*, where the S variant exhibited a well-defined and large ETZ inside phagosomes, while an R variant carrying a single non-synonymous point mutation in the gene encoding MmpL4a, another determinant of GPL synthesis and transport, had almost no visible ETZ (Bernut et al., 2016). Strikingly, at later points of infection with only the S variant of *M. abscessus* we observed disruptions in the membranes of phagosomes containing bacteria, indicating that *M. abscessus* like the pathogenic SGM has the ability to induce phagosome-cytosol communications (Stamm et al., 2003; van der Wel et al., 2007; Simeone et al., 2012). Curiously, while it was shown that the ability of *M. tuberculosis* and *M. marinum* to escape the phagosome into the cytosol was strictly dependent on the presence of the type VII secretion system ESX1 (Simeone et al., 2012), *M. abscessus* genomes only encode ESX3 and ESX4 type VII secretion systems (Dumas et al., 2016), either of which have yet to be implicated in mycobacterial pathogenicity.

Together, these observations point to different infection programs followed by *M. abscessus* S and R variants. The S variant is readily phagocytosed by macrophages without severely impacting the survival of the macrophages, while the R variant, phagocytosis of which is detrimental to macrophage viability, prefers an extracellular lifestyle typified by cording as a major immune evasive mechanism whereby phagocytosis is physically impeded. We recently also showed that intracellular *M. abscessus* shares the ability with *M. tuberculosis* to use the abundant host lipid triacylglycerol (TAG) found in foamy macrophages as a rich carbon nutrient (Viljoen et al., 2016), a characteristic which is believed to contribute to the ability of *M. tuberculosis* to cause a latent infection (Peyron et al., 2008). Indeed, evidence exist that *M. abscessus*, like *M. tuberculosis*, can cause asymptomatic infection lasting for years before a full-fledged acute infection emerges (Moore and Frerichs, 1953; Cullen et al., 2000). One could, therefore, speculate that the S variant may act as a reservoir of live bacilli during this latent period of infection, and once mutations in the GPL locus occur allowing the emergence of R bacilli, a much more aggressive lifestyle is adopted by the bacteria, characterized by acute disease. Indeed, clinical evidence points out toward the S variant being the invasive form probably causing initial infection, while the R form which later emerges causes more severe forms of the disease (Catherinot et al., 2009).

### Inhibition of *M. abscessus* mycolic acid transport

In two recent reports, we detail our discovery of novel unrelated non-toxic chemotypes that efficiently inhibit both extracellular and intracellular *M. abscessus* populations through inhibition of highly essential mycolic acid transport (Figure 1). Mycolic acids are extremely large fatty acids consisting of a long β-hydroxy fatty acid chain (C_60−90_) with a shorter α-alkyl branch (C_24−26_) and lend to the mycobacterial cell wall its renowned hydrophobicity and impermeability to extraneous compounds like antibiotics. These essential fatty acids are biosynthesized for the larger part within the mycobacterial cytosol through the joint actions of fatty acid synthase I (FasI), the FasII complex, an acyl-AMP ligase and a polyketide synthase, the last step resulting in the transacylation of trehalose with the α-alkyl β-ketoacyl mycolic acid to produce trehalose monomycolate (TMM) (Quémard, 2016). After an acetylation step of TMM, the molecule is transferred to the periplasmic space *via* the essential mycolic acid transporter, MmpL3 (Yamaryo-Botte et al., 2015; Xu et al., 2017). Once inside the periplasm, TMM is probably deacetylated by an unidentified enzyme. In the mycomembrane, the antigen 85 enzyme complex uses TMM as substrate to transfer the mycolic acids onto arabinogalactan as well as to produce trehalose dimycolate (TDM), also known as cord factor (Belisle et al., 1997).

A surprisingly large number of recent chemical hits against *M. tuberculosis* have been assigned to target MmpL3 activity leading to the view that this protein could represent the Achilles' heel of mycobacteria (Nataraj et al., 2015). We also serendipitously identified a novel piperidinol derivative potently inhibiting MmpL3 in *M. abscessus* (compound 1, Figure 1), when we performed a cross screen of a library of compounds with known anti-*M. tuberculosis* activity against *M. abscessus* (Dupont et al., 2016). More recently, we evaluated a structurally unrelated chemotype class, the indole-2-carboxamides (exemplified by compounds 2 and 3, Figure 1) previously shown as potent anti-tubercular compounds (Lun et al., 2013; Onajole et al., 2013; Stec et al., 2016), for their activity against *M. abscessus* and found that they too potently inhibited mycolic acid transport (Kozikowski et al., 2017). Sequencing analysis of the *mmpL3* gene in spontaneous resistant mutants selected on either of the compound classes revealed a common Ala309Pro substitution in transmembrane domain 5. Over-expression of MmpL3 carrying the Ala309Pro mutation in *M. abscessus* wild-type bacteria conferred high level resistance to both the piperidinol-based compound and indole-2-carboxamides, confirming their target. Modeling of the three-dimensional structure of MmpL3 revealed a large cavity formed by transmembrane helices 5, 7, 8, 9, and 10 where the piperidinol-based compound could potentially bind (Dupont et al., 2016). Importantly, both the piperidinol-based and indole-2-carboxamide compounds showed good activity against *M. abscessus* both extracellularly (Table 1) and intracellularly in macrophages and improved the survival of zebrafish embryos infected with the R strain in the case of the piperidinol-based compound (Dupont et al., 2016). Not only were these compounds found to be non-toxic toward the HepG2 human cell line (compound 1) (Ballell et al., 2013) and Vero cells (compounds 2 and 3) (Kozikowski et al., 2017), but they were also active at lower concentrations than the drugs that are currently being used to treat *M. abscessus* in the clinical setting (Table 1). In addition, both chemotypes were equally efficient against the S and R variants of a wide panel of clinical strains of all three *M. abscessus* subspecies isolated from CF and non-CF patients. The fact that these non-toxic compounds worked efficiently inside and outside macrophages, highlights their potential for development into a new class of antibiotics active against *M. abscessus*. From a medicinal chemistry perspective and for future drug development, these compounds are easy to prepare and early studies on the indole-2-carboxamides indicated that they show reasonably good ADME properties despite their high lipophilicity (Onajole et al., 2013; Stec et al., 2016; Kozikowski et al., 2017).

**Table 1:** MIC of a piperidinol derivative, indole-2-carboxamides and drugs used in the clinic against *M. abscessus* CIP104536^T^ S and R variants.

**Compound**	**MIC (μg/ml)**		**References**
	**S**	**R**	
1 (piperidinol derivative)	0.125	0.125	Dupont et al., 2016
2 (indole-2-carboxamide derivative)	0.125	0.125	Kozikowski et al., 2017
3 (indole-2-carboxamide derivative)	0.125	0.125	Kozikowski et al., 2017
Clarithromycin	32	64	Dupont et al., 2016
Amikacin	32	16	Dupont et al., 2016
Cefoxitin	32	64	Dupont et al., 2016
Imipenem	4	2	Dupont et al., 2016

## Conclusions

While we are starting to understand how *M. abscessus* follows a different infection program to the other mycobacteria that cause lung pathology, favoring an extracellular lifestyle during exacerbation of the disease, many questions remain unsolved. Although several reports documented that infections with the three *M. abscessus* subspecies can lead to different clinical outcomes (Lee et al., 2015; Jeong et al., 2017; Park et al., 2017), detailed studies on the physiology of *M. massiliense* and *M. bolletii* are sparse, apart from one study showing similar infection outcomes for the three subspecies in zebrafish embryos (Bernut et al., 2014). Therefore, comparative cellular biology studies to understand their lifestyles within macrophages are warranted. Although being highly similar at a genetic level, such data could be valuable especially to pharmacodynamics studies, which should consider the relative importance of an intracellular lifestyle of a pathogen. While it is clear that the R form prefers an extracellular lifestyle, it is surprising that the S variant, which appears to reside inside macrophages, characteristic of the intracellular lifestyle of SGM, persists within these cells. Due to its spectacular natural multidrug resistance, the list of available antibiotics to treat *M. abscessus* infections is short. Most antitubercular drugs, including isoniazid, that work through inhibiting mycolic acid biosynthesis, are not active against *M. abscessus*. However, efficient inhibition of *M. abscessus* growth through inhibition of mycolic acid transport by two sets of non-toxic and unrelated chemotypes shows that promise exists for the future development of chemotherapies against this pathogen. Future work will be performed to assess the efficacy of these compounds in *M. abscessus* animal models.

## Author contributions

AV contributed to writing the manuscript and designed the figure. JH contributed to writing the manuscript. OO contributed to writing the manuscript. JS contributed to writing the manuscript. AK contributed to writing the manuscript. LK contributed to writing the manuscript and designed the figure.

### Conflict of interest statement

The authors declare that the research was conducted in the absence of any commercial or financial relationships that could be construed as a potential conflict of interest.
